# A Sparse Mixture-of-Experts Model With Screening of Genetic Associations to Guide Disease Subtyping

**DOI:** 10.3389/fgene.2022.859462

**Published:** 2022-06-06

**Authors:** Marie Courbariaux, Kylliann De Santiago, Cyril Dalmasso, Fabrice Danjou, Samir Bekadar, Jean-Christophe Corvol, Maria Martinez, Marie Szafranski, Christophe Ambroise

**Affiliations:** ^1^ Université Paris-Saclay, CNRS, Université d’Évry, Laboratoire de Mathématiques et Modélisation d’Évry, Évry-Courcouronnes, France; ^2^ Sorbonne Université, Paris Brain Institute–ICM, Inserm, CNRS, Assistance Publique Hôpitaux de Paris, Pitié-Salpêtrière Hospital, Department of Neurology, Paris, France; ^3^ Institut de Recherche en Santé Digestive, Inserm, CHU Purpan, Toulouse, France; ^4^ ENSIIE, Évry-Courcouronnes, France

**Keywords:** mixture of experts model, disease subtyping, clinical data, longitudinal data, genotyping, high dimension, variable selection, Parkinson’s disease

## Abstract

**Motivation:** Identifying new genetic associations in non-Mendelian complex diseases is an increasingly difficult challenge. These diseases sometimes appear to have a significant component of heritability requiring explanation, and this missing heritability may be due to the existence of subtypes involving different genetic factors. Taking genetic information into account in clinical trials might potentially have a role in guiding the process of subtyping a complex disease. Most methods dealing with multiple sources of information rely on data transformation, and in disease subtyping, the two main strategies used are 1) the clustering of clinical data followed by posterior genetic analysis and 2) the concomitant clustering of clinical and genetic variables. Both of these strategies have limitations that we propose to address.

**Contribution:** This work proposes an original method for disease subtyping on the basis of both longitudinal clinical variables and high-dimensional genetic markers *via* a sparse mixture-of-regressions model. The added value of our approach lies in its interpretability in relation to two aspects. First, our model links both clinical and genetic data with regard to their initial nature (i.e., without transformation) and does not require post-processing where the original information is accessed a second time to interpret the subtypes. Second, it can address large-scale problems because of a variable selection step that is used to discard genetic variables that may not be relevant for subtyping.

**Results:** The proposed method was validated on simulations. A dataset from a cohort of Parkinson’s disease patients was also analyzed. Several subtypes of the disease and genetic variants that potentially have a role in this typology were identified.

**Software availability:** The R code for the proposed method, named DiSuGen, and a tutorial are available for download (see the references).

## 1 Introduction

Known genetic markers in complex diseases usually account for only a part of calculated heritability. One possible explanation is that these complex diseases have different subtypes with different genetic factors. The identification of subtypes can nowadays draw upon large heterogeneous datasets, including patient follow-up and genotyping data.

When clinical and genomic information is available, subtyping can adopt either of two approaches: 1) the clustering of clinical data with a posterior genetic analysis, or 2) the concomitant clustering of clinical and genomic data. We will discuss the pros and cons of these two approaches in Section 2.

### 1.1 Contributions

In this work, we sketch a third way at the crossroads between the two approaches mentioned previously. This alternative approach consists in clustering the clinical variables by estimating a multinomial logistic regression model whose weights depend on the genetic variables. The model reflects the longitudinal nature of the clinical data and addresses the high dimensionality of the problem *via* a sparse constraint on the parameters involved in the logistic weights.

### 1.2 Organization of the Article

Section 2 gives an overview of different strategies that may be used for disease subtyping where there are different sources of information. Section 3 proposes a framework related to mixture-of-experts models, for clustering clinical longitudinal data guided by genetic markers. Section 4 describes our proposed algorithm and its implementation in a high-dimensionality setting. Section 5 provides an illustration of our approach using numerical simulations, and Section 6 gives an analysis of a cohort of patients with Parkinson’s disease.

## 2 Disease Subtyping With Multiple Sources of Information

In this section, we briefly describe the various approaches used for clustering where there are different sources of data, focusing in particular on methods for disease subtyping with multiple information sources.

### 2.1 Clustering of Clinical Data With Posterior Genetic Analysis

As outlined in the following, this is a two-step approach involving 1) disease subtyping based on clinical data, followed by 2) an analysis of the genetic associations in each subtype.

#### 2.1.1 Clustering of Clinical Data

The data often come from clinical follow-ups, and as such are generally longitudinal in nature. A review of clustering methods suitable for functional data, including longitudinal data, is discussed by Jacques and Preda (2014), with the following categorization:

• *Methods with a filtering step* consist in characterizing the curves in terms of a few descriptors such as their slope and intercept, and then clustering on those descriptors.
• *Non-parametric methods*, such as *K*-means, with distance metrics adapted to longitudinal data.
• Finally, *model-based methods* appear to be the most suitable methods for the kind of short longitudinal data with numerous missing values that often arise from medical follow-ups. An overview of the approaches and tools devoted to *mixture models* for longitudinal data has been proposed by van der Nest et al. (2020).

*Remark.* In this work, we focus on *mixtures of experts*, a specific category of *mixture models* (Section 3.1).

#### 2.1.2 Analysis of Clinical Clusters With Genomics

Following the clustering, this second step seeks to exhibit genetic associations underlying the clusters. One way of doing this would be to use the clusters as phenotypes in standard GWAS approaches that usually involve statistical procedures based on (multiple) hypothesis testing (Bush and Moore, 2012; Hayes, 2013). Another way would be to resort to classical supervised methods, such as (multinomial) logistic regression, with a feature selection procedure (Ma and Huang, 2008).

#### 2.1.3 Limitation

Since the genetic analysis takes place only after the clustering of clinical data has been completed, the clustering step makes no reference to the genomic data. As a consequence, there can be no certainty regarding an association between the genomic information and the clinical clusters. Also, most sparse model-based clustering methods for high-dimensional functional or longitudinal data need to resort to dimensionality reduction techniques such as PCA or SVD, which are effective but present barriers to interpretation.

### 2.2 Concomitant Clustering of Clinical and Genomic Data

Concomitant clustering using both clinical and genomic data represents an attractive alternative to the two-step approach described previously. However, a large number of variables may be present, meaning that feature- or variable-selection strategies are required to solve the problem.

#### 2.2.1 Multi-View Clustering

This framework, developed within the machine learning community, provides a number of popular methods for solving problems with different feature sets. The survey by Fu et al. (2020) groups these methods into three categories.

• *Graph-based methods* combine different views according to their respective importance and then generally resort to spectral clustering algorithms.
• *Space-learning-based methods* are designed to construct a new learning space using the most representative characteristic of each view to enhance clustering.
• *Binary-code-learning-based methods* encode original data as binary features using mapping and reduction techniques to reduce computation time and memory use.

We also need to mention the *Multiple Kernel Learning* framework for clustering (Zhao et al., 2009), which corresponds to another kind of multi-view learning. In particular, Mariette and Villa-Vialaneix (2017) proposed (consensus) meta-kernels for aggregating different sources of information while preserving the original topology of the data. Among the various works devoted to disease subtyping using clinical and genomic information, those that come within the scope of multi-view clustering use *space-learning-based methods* with dimensionality reduction approaches. Sun et al. (2014) propose a multi-view co-clustering method based on Sparse Singular Value Decomposition (Lee et al., 2010). Sun et al. (2015) build on this work, providing convergence guarantees using the proximal alternating linearized minimization algorithm proposed by Bolte et al. (2014).

#### 2.2.2 Integrative Clustering

In cancer research, a variety of statistical methodologies have emerged for analyzing data coming from different sources, generally multiple omics data, within the field of *integrative genomics* (Kristensen et al., 2014). The philosophy underlying these methodologies is closely related to multi-view learning. Huang et al. (2017) present a review of multi-omics integration tools. We must also mention *mixOmics* (Rohart et al., 2017), which proposes various sparse multivariate methods for exploring multiple omics datasets. More specifically, integrative clustering may be built on model-based approaches such as in the representative work by Shen et al. (2009) and Shen et al. (2010). The *iCluster* method uses a latent variable model to connect multiple data types. The optimization of a penalized log-likelihood involves a process of dimensionality reduction on the representation of the original data that iteratively alternates with several extensions of *iCluster* using penalties inducing different types of sparsity which have been proposed since (Shen et al., 2013; Kim et al., 2017). Finally, *PINSPlus* (Nguyen et al., 2018), to identify subtypes across different views, uses a perturbation scheme applied to each source of data to define stable clusters, before merging results using different algorithms to construct a similarity matrix based on the overall connectivity of the patients.

#### 2.2.3 Limitations

Concomitant approaches can be suitable for solving problems related to clinical and genomic datasets. However, none of these approaches provides an explicit recipe for dealing with heterogeneous data.^1^ In particular, the longitudinal aspect is not taken into account in these kinds of approaches. In addition, most methods require new representations derived from the original space. Distorting the initial information may significantly complicate the posterior validation of the extracted features. The inherent limitation of methods based on dimensionality reduction was referred to previously. An additional difficulty arises with methods based on similarity matrices, such as kernel methods that implicitly map the data in a new feature space, since these methods require a pre-image problem to be solved for features to be approximated and, where possible, interpreted.

## 3 Mixtures of Regressions With Clinical and Genomic Data

To take advantage of both the clinical and the genomic information, the two datasets can be used simultaneously *via* a mixture model. Mixtures of experts provide an elegant framework for including concomitant variables as secondary information alongside subtype data (Gormley et al., 2019). This section starts with a description of mixture-of-experts models, with a view to clarify the links between this framework and the approach that we are proposing.

### 3.1 Mixture-of-Experts Models

Let **Y** be a matrix of *N* observed outcomes represented by variables *v* ∈ {1⋯*V*} such that **y**_*i*_ = (*y*_*i*1_, … , *y*_*iv*_, … , *y*_*iV*_), for *i* ∈ {1⋯*N*}. These observations come from a population of *K* components. **z** = (*z*_1_, … , *z*_*i*_, … , *z*_*N*_) is the component membership vector where *z*_*i*_ ∈ {1⋯*K*}, and **Z** is the corresponding indicator matrix such that **z**_*i*_ ∈ {0,1}^*K*^, with *z*_*ik*_ = 1 if the observation *i* belongs to the *k* ^th^ component and *z*_*ik*′_ = 0, otherwise (*∀k*′ ≠ *k*). A matrix **G** of *N* concomitant data represented by variables *ℓ* ∈ {1⋯*L*} is also available, with **g**_*i*_ = (*g*_*i*1_, … , *g*_*iℓ*_, … , *g*_*iL*_), for *i* ∈ {1⋯*N*}. The random vectors corresponding to these representations are respectively denoted by Y, Z, and G.

*Remark.* To lighten notations, the range of indexes will often be omitted, in which case the ranges of indexes *i*, *v*, *ℓ,* and *k* (or *k*′) will be as defined previously.

Using the terminology in Gormley (21, Section 2.3), we are interested in *simple mixture-of-experts models* where the outcome data distribution depends on the latent component membership, which itself depends on the concomitant variables, such that $P\left(y_{i},z_{i}\;|\;g_{i}\right)=f_{z_{i}}\left(y_{i}\;;\;\Theta_{z_{i}}\left(g_{i}\right)\right)\;\eta_{z_{i}}\left(g_{i}\right)$ , with

$$y_{i}\;|\;g_{i},z_{i}=k∼f_{k}\left(y_{i}\;;\;\Theta_{k}\left(g_{i}\right)\right)\;,$$ (1a)

$$\text{and}\;P\left(z_{i}=k\;|\;g_{i}\right)=\eta_{k}\left(g_{i}\right),$$ (1b)

where Θ_*k*_(⋅) is the set of parameters of the *k* ^th^ component density function *f*_*k*_(⋅; Θ_*k*_(⋅)), that is, the *k* ^th^ expert, and *η*_*k*_(⋅) the probability weight related to the *k* ^th^ expert.

### 3.2 Proposed Approach

Based on the previously described framework, we propose a mixture-of-regressions model over time for disease subtyping, where patient symptoms are recorded from their follow-up along with genetic markers as concomitant variables. Each cluster thus describes the evaluation of the symptoms over time and is simultaneously linked to a set of genetic markers.

#### 3.2.1 Specificity

Our model is designed to take into account the longitudinal aspect of the clinical data and the high-dimensional nature of the genetic data. **Y** comprises observed values of clinical variables over a series of follow-up visits indexed by *j*. The *v*th clinical variable observed during the *j* ^th^ visit of patient *i* is denoted $y_{iv_{\left(j\right)}}$ . Also, the number of variables in the genetic data **G** may be of the order of a few million after genotype imputation, so that dedicated metrics [such as CADD (Rentzsch et al., 2018), used in our explanation concerning Parkinson’s disease] or more general elimination techniques such as screening rules [see (Ndiaye et al., 2017) for instance] may still be required beforehand. Note that even where this kind of prior processing occurs, we remain in a configuration where *N* ≪ *L*.

#### 3.2.2 Model

To connect our proposal with the mixture of experts given previously in Eqs 1a, 1b, we characterize the problem as

$$y_{iv_{\left(j\right)}}\;|\;g_{i},z_{i}=k∼f_{k}\left(y_{iv_{\left(j\right)}}\;;\;\left\{\alpha_{vk},\sigma_{vk}\right\}\right)\;,$$ (2a)

$$\text{and}\;P\left(z_{i}=k\;|\;g_{i}\right)=\eta_{k}\left(g_{i}\;;\;\omega_{k}\right),$$ (2b)

defining the following regression model with logistic weights:

$$\left(y_{iv_{\left(j\right)}}\;|\;z_{i}=k\right)=\overset{P}{\underset{p\;=\;0}{\sum }}\alpha_{\mathrm{vkp}}\;t_{ij}^{\;p}+\sigma_{vk}\;\varepsilon_{iv_{\left(j\right)}},$$ (3a)

$$\text{such that}\;f_{k}\left(y_{iv_{\left(j\right)}}\;;\;\left\{\alpha_{vk},\sigma_{vk}\right\}\right)∼N\left(\overset{P}{\underset{p\;=\;0}{\sum }}\alpha_{\mathrm{vkp}}\;t_{ij}^{\;p},\;\sigma_{vk}^{2}\right),$$ (3b)

$$\text{and}\;\eta_{k}\left(g_{i}\;;\;\omega_{k}\right)=\frac{\exp\left(\omega_{k0}+\omega_{k}^{⊺}g_{i}\right)}{\underset{k^{\prime }}{\sum }\exp\left(\omega_{k^{\prime }0}+\omega_{k^{\prime }}^{⊺}g_{i}\right)},$$ (3c)

where

• *t*_*ij*_ is the time metric, which might, for example, be the patient’s age or time since the disease was first diagnosed, for the patient *i* at their *j*th follow-up visit,
• *p* ∈ {0⋯*P*} is the polynomial degree considered in the regression (*P* = 2 is generally sufficient),
• {*α*_*vkp*_}, {*σ*_*vk*_}, and {***ω***_*k*_} are parameters or vectors to be estimated, with {*ω*_1*ℓ*_} = 0 for the sake of identifiability,
• $\varepsilon_{iv_{\left(j\right)}}\underset{iid}{∼}N\left(0,1\right)$ , implies some conditional independence assumptions between variables, patients, and visits when the class is known. The clinical variables are chosen to be as independent as possible, the correlation between individuals should essentially come from a similar typology of the disease, and finally, the remaining time correlation after the polynomial regression is expected to be poor. If the Gaussian hypothesis does not apply to the variable *v*, Poisson or logistic regression may be considered instead, with no substantial additional cost.

The longitudinal aspect is taken into account by assuming for each cluster the existence of typical temporal trajectories, described by a polynomial regression of clinical variables over time, around which the patients’ symptoms evolve. These are assumed to fully summarize the temporal evolution of each patient. According to this model, there is no residual intra-patient correlation conditional on the trajectory followed (requiring knowledge of the cluster). The modeling of posterior probabilities *via* logistic regression allows concomitant variables, such as genetic data, to subtly influence the subtyping.

#### 3.2.3 Model Selection

We combine two model selection strategies to select the hyperparameters involved in the mixture. The first of these is the Bayesian Information Criterion (BIC), which is widely used within the research community to select *K*, the most appropriate number of subtypes, and *P*, the polynomial degrees in the main regressions. Also, as discussed previously, we suspect that many variables *ℓ* from **G** will have little or no influence on disease phenomenology. A Lasso penalization is therefore applied on the coefficients {***ω***_*k*_}, *∀k*, to select those that have the most relevance in the subtyping. More details about this aspect are given in Section 4.

## 4 Expectation-Maximization Algorithm With Integrated Lasso Inference

The inference of this kind of model with latent variables, here {*z*_*ik*_}, is traditionally done with the aid of an Expectation-Maximization algorithm [EM algorithm, (Dempster et al., 1977)]. We use a modified version of this algorithm with a Lasso-type penalized likelihood instead of classical likelihood.

### 4.1 Expectation-Maximization Algorithm

The (*q* + 1)th iteration of the modified EM algorithm maximizes the expected and penalized complete-data log-likelihood L(Y|G,Z;Θ={α,σ,ω})−P(ω) which reads

$$\underset{i}{\sum }\underset{k}{\sum }z_{ik}\left[\log\left[\eta_{k}\left(g_{i}\;;\;\omega_{k}\right)\right]+\underset{v}{\sum }\underset{j}{\sum }\log\left[f_{k}\left(y_{iv_{\left(j\right)}}\;;\;\left\{\alpha_{vk},\sigma_{vk}\right\}\right)\right]\right]-\lambda \underset{k}{\sum }\|\omega_{k}{\|}_{1},$$

where *λ* > 0 controls the amount of sparsity applied on the *ℓ*_1_ norm of ***ω***_*k*_ and where *η*_*k*_(⋅; ⋅) and *f*_*k*_(⋅; ⋅) are defined as in Eqs 3a,3b,3c.

To maximize the expected and penalized complete-data log-likelihood, each iteration is separated into an expectation step (E) followed by a maximization step (M).

• At step E of the (*q* + 1)th iteration, posterior weights are updated as follows:

$$\begin{array}{ll} \tau_{ik}^{\left(q+1\right)} & =E\left[z_{ik}|Y=y_{i},g_{i};\Theta^{\left(q\right)}\right]\\ & =\frac{\eta_{k}\left(g_{i}\;;\;\omega_{k}^{\left(q\right)}\right)\underset{v}{\prod }\underset{j}{\prod }f_{k}\left(y_{iv_{\left(j\right)}}\;;\;\left\{\alpha_{vk}^{\left(q\right)},\sigma_{vk}^{\left(q\right)}\right\}\right)}{\underset{k^{\prime }}{\sum }\eta_{k^{\prime }}\left(g_{i}\;;\;\omega_{k^{\prime }}^{\left(q\right)}\right)\underset{v}{\prod }\underset{j}{\prod }f_{k^{\prime }}\left(y_{iv_{\left(j\right)}}\;;\;\left\{\alpha_{vk^{\prime }}^{\left(q\right)},\sigma_{vk^{\prime }}^{\left(q\right)}\right\}\right)}. \end{array}$$

• At step M of the (*q* + 1)th iteration, parameters are updated as follows:

$$\Theta^{\left(q+1\right)}=\underset{\Theta }{argmax}\underset{i}{\sum }\underset{k}{\sum }\tau_{ik}^{\left(q+1\right)}\left[\log\left[\eta_{k}\left(g_{i}\;;\;\omega_{k}\right)\right]+\underset{v}{\sum }\underset{j}{\sum }\log\left[f_{k}\left(y_{iv_{\left(j\right)}}\;;\;\left\{\alpha_{vk},\sigma_{vk}\right\}\right)\right]\right]-\lambda \underset{k}{\sum }\|\omega_{k}{\|}_{1}.$$

The maximization with regard to parameters {***α***, ***σ***} presents no difficulty (Supplementary Material). However, there is no closed formula that may be used for updating the logistic weights parameters. The term to be maximized with respect to {***ω***} at iteration (*q* + 1) of the EM algorithm is

$$\frac{1}{N}\underset{i}{\sum }\underset{k}{\sum }\tau_{ik}^{\left(q+1\right)}\log\left[\eta_{k}\left(g_{i}\;;\;\omega_{k}\right)\right]-\lambda \underset{k}{\sum }\|\omega_{k}{\|}_{1}.$$ (4)

This maximization problem corresponds to the multinomial logistic regression problem with a *ℓ*_1_ penalty, which can be solved using a proximal-Newton approach (Hastie et al., 2015). ^2^

### 4.2 Initialization and Variable Selection in Practice

The EM algorithm is subject to local optima. To address this classical problem and to provide stability and improve robustness, we perform a variety of initializations and retain the initialization that yields the lowest BIC.

Strategies commonly used for selecting the hyperparameter *λ* are based on adjusted information criterion [ (Chen and Chen, 2012) for General Linear Models or (Fop and Murphy, 2018) for a more general overview]. In an original approach, Yi and Caramanis (2015) proposed optimizing the hyperparameter *λ* *via* an iterative scheme over successive M steps, and showed local convergence properties in the high dimensional setting.

In this work, we use an alternative adopted by Mortier et al. (2015), where *λ* is chosen within the M step by cross-validation such that the likelihood of the multinomial logistic model (4) is maximized. The simulation study described in Section 5 showed that proceeding with this selection at every M step of the EM algorithm does not compromise convergence.

Finally, to avoid (negative) bias due to the penalization in the parameter estimation, we re-estimate the selected {***ω***} parameters at the end of the EM algorithm to obtain the maximum likelihood estimates, which is the usual practice [(Hastie et al., 2009), p. 91].

### 4.3 Implementation

The implementation of the method proposed in this study, which we have named DiSuGen, and an R Markdown tutorial are available for download (Courbariaux et al., 2020). We build on the FlexMix R package (Grun and Leisch, 2008) which proposes an EM algorithm suitable for multinomial logistic mixture models. To implement our method, we developed an adapted concomitant variable driver making use of glmnet within FlexMix. For a faster convergence, we resort in practice to a Classification EM (CEM) algorithm in which $\tau_{ik}^{\left(q+1\right)}$ are replaced by the indicator variables $z_{ik}^{\left(q+1\right)}$ (Celeux and Govaert, 1992).

## 5 Numerical Tests Using Artificial Data

We used artificial data to test our proposed estimation and model selection procedures. These simulations are designed to assess the ability of the CEM algorithm both to produce a good estimation of the parameters and to obtain the appropriate model. The methodology is given in detail as follows.

### 5.1 Data Generation

Artificial data are simulated according to the model (3) with *N* = 396 patients, *V* = 4 clinical variables, *K* = 3 clusters, *P* = 1 polynomial degree in the regression, three follow-up visits per patient with times *t*_*ij*_ randomly ranging from 10 to 410 days for the first visit, from 1,800 to 2,200 days for the second, and from 3,600 to 4,000 days for the third. Also, *L* = 2,657 genetic markers are simulated with only 10 having an influence on the clustering such that ***ω***_*k*{*ℓ*}_ is ***ω***_2{2,3,4}_ = ***ω***_3{5,6,7}_ = 2, ***ω***_2{5,6,7}_ = −1, and ***ω***_3{1,8,9,10}_ = −2. For the sake of consistency with the study presented in Section 6, the genetic markers come from the Parkinson’s disease genetic data, and the parameters {***α***, ***σ***} are chosen to be realistic with regard to the Parkinson’s disease clinical data.

### 5.2 Protocol

For each simulation, the proposed CEM algorithm is run with *K* = 3 clusters and a Lasso penalty. The estimation is initialized with 10 sets of starting values corresponding to 10 random assignments into *K* = 3 clusters, and the set of values that gives the lowest BIC is retained. The experiment is repeated 100 times. To assess the performance of our method, we compare many different methods:

• The *integrative* method is the one described in this study, which uses both clinical and genetic data and estimates the parameters {***α***, ***σ***} and {***ω***}, and the subtypes **z**.
• The *oracle integrative* and *semi-oracle integrative* methods also use both clinical and genetic data to estimate the subtypes **z**. The *oracle integrative* uses all parameters of the model set to their true values. The *semi-oracle integrative* method sets only the parameters {***ω***} to their true values. This allows us to check to what extent our method correctly subtypes the data and estimates the parameters relating to clinical variables (semi-oracle) and genetic variables (oracle).
• The *2-step* method does not use genetic information in the clustering process to estimate the parameters {***α***, ***σ***}, and clusters have constant weights, that is, $P\left(z_{ik}=1\right)=\pi_{k}$ . In this case, the Lasso-penalized multinomial logistic regression is performed afterward to give genetic association results. This allows us to assess the benefit of including genetic information in the clustering process at the same time that the clinical parameters are estimated.
• The *oracle 2-step* method is identical to the *2-step* method, except that the parameters {***α***, ***σ***} are set to their true values.
• Where possible, the proposed method is also compared with the *K*-means method, which corresponds to a simple Gaussian mixture model with identical proportions and identical standard deviations in all clusters. For this purpose, we use a *K*-means method adapted to longitudinal data implemented in the R package kml3d (Genolini et al., 2015).

### 5.3 Results

#### 5.3.1 Clustering Ability

**FIGURE 1:**
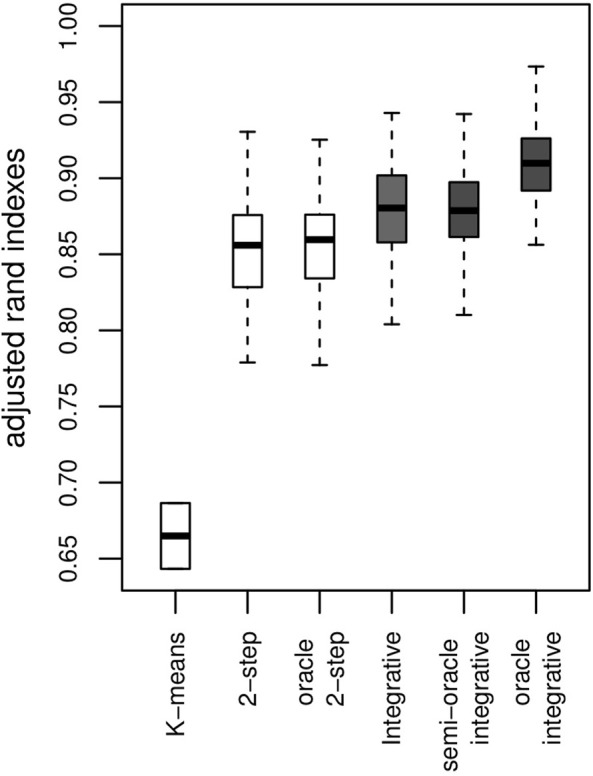
Results on artificial data over 100 simulations. ARI with a *K*-means algorithm, the *2-step* method (no use of genetic information) and the integrative method (use of genetic information) and their corresponding oracles. The *y*-axis represents the ARI score (the higher the better).

The Adjusted Rand Index [ARI, (Rand, 1971; Hubert and Arabie, 1985)] is computed for each simulation to check that the estimated clusters are close to those that are being simulated (a higher ARI score is more desirable). The results for our proposed method are shown in the boxplot for the *integrative* method in Figure 1. Most clusters are well identified. Where no use is made of genetic information within the clustering, ARI values obtained by the algorithm are lower. As we might expect, the algorithm making use of genetic data achieves better clustering, as shown by the *oracle integrative* results. This improvement in cluster prediction is not, however, because of a better estimation of the parameters {***ω***}, as shown by the *semi-oracle integrative* results. Finally, the *K*-means algorithm is less effective in recovering the underlying classification, with ARI values all between 0.6 and 0.7. This was expected, since the differences between the clusters partly lie in the variances of the variables. Moreover, the *K*-means method does not address the times of the follow-up visits, but only their sequence numbers.

#### 5.3.2 Parameter Estimation Ability

**FIGURE 2:**
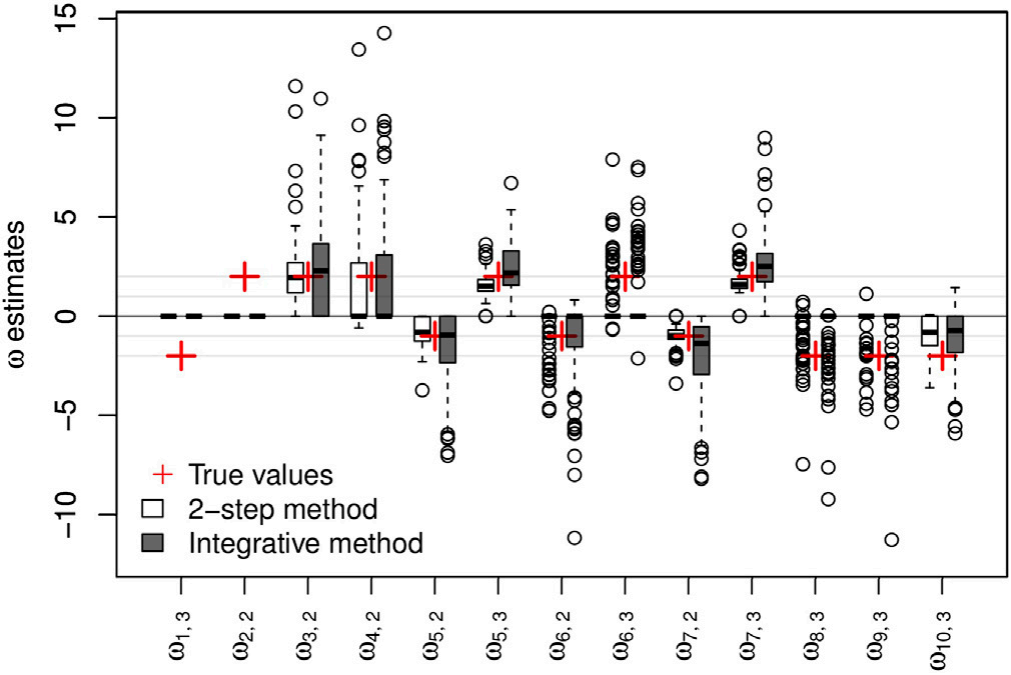
Results on artificial data over 100 simulations. Non-negative {***ω***} parameter estimates and their respective true values. The *y*-axis represents the values of the estimates.

The parameters of the main regressions are estimated accurately and with biases close to 0 irrespective of the clinical variable considered and the approach used (*2-step* or *integrative*). Taking genetic information into account does not appear to offer any great improvement in the estimation of these parameters. Regarding the logistic regression parameters, the sign of the estimated parameters is mostly reflected correctly in the two approaches, as shown in Figure 2.

#### 5.3.3 Variable Selection Within the Logistic Regression

**FIGURE 3:**
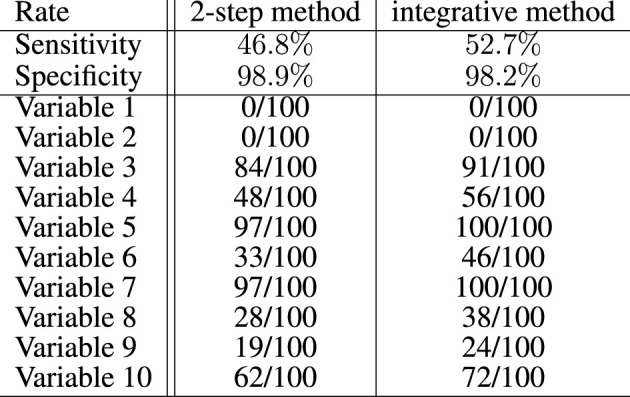
Global sensitivity and specificity of the integrative method compared with the 2-step method for the selection of the genetic variables in the artificial data experiment with 100 simulations. Among 2,657 variables, 10 had to be selected. The number of times these variables have been selected over the 100 simulations is also specified for both methods.

Figure 3 summarizes the results of the proposed Lasso selection procedure with regard to the genetic variables. The sensitivity of the proposed *integrative* approach (52.7%) is higher than that of the equivalent *2-step* method (46.8%). It was computed globally over the 100 simulations and for the 10 active genetic variables. With both methods, the selection rates of 8 of the 10 active genetic variables are notably higher than the selection rates of the other variables, which indicates that the selection method performs well. The two remaining active markers do not vary between patients, and could therefore be replaced by any variant with a low variation. The specificity of both approaches is good, with a slightly better result for the *2-step* method (98.9%, vs. 98.2% for the *integrative* approach). This result is an overall result computed over the 100 simulations and for the 2,647 inactive genetic variables. Selection performance decreases, as expected, the closer the parameters {***ω***} are set to zero (data not shown).

#### 5.3.4 Selection Ability of the Model

**FIGURE 4:**
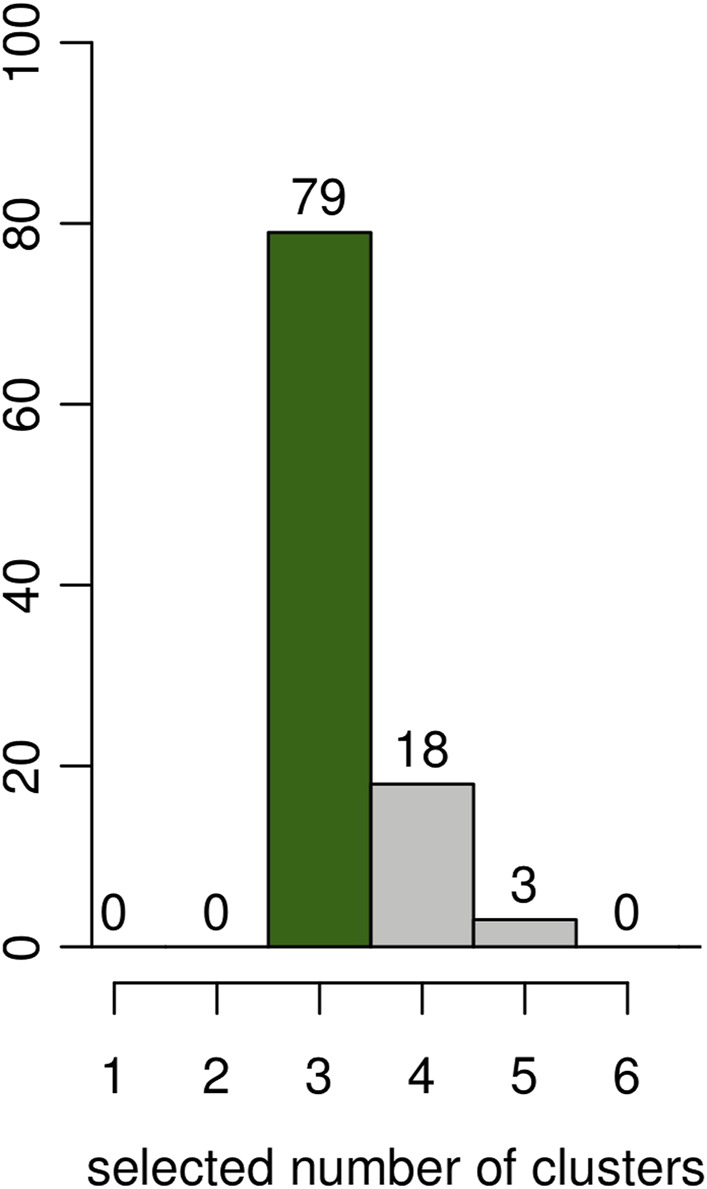
Results on artificial data over 100 simulations. Estimated number of clusters according to the BIC. The *y*-axis represents the number of simulations for the number of clusters selected in the *x*-axis.

An additional simulation was done to evaluate the capacity of the BIC (computed as described in Section 3) to select the correct number of clusters (*K* = 3) on the same 100 simulated datasets. The results are shown as the histogram in Figure 4. The correct number of clusters is selected 79 times out of 100.

## 6 Demonstration Using Parkinson’s Disease Subtyping

We applied our proposed method to PD subtyping. PD is known to have several subtypes, and there are a number of relevant studies, including the study by Lewis et al. (2005).

### 6.1 Data Description

The data on which we applied our method come from the DIG-PD cohort (Corvol et al., 2018) comprising 396 genotyped adults with a recent PD onset (diagnosed less than 6 years before the beginning of the study).

#### 6.1.1 Clinical Data

Clinical data were collected at inclusion and then at yearly clinical follow-ups between 1 and 7 years. They include scores evaluating the progression of the disease. Two of these scores are taken to be representatives of the evolution of the disease, namely *UPDRS III* (Section III of the Unified PD Rating Scale, a motor examination), and *MMSE* (the score from the Mini-Mental Status Examination tool kit, an evaluation of cognitive impairment). The higher UPDRS III and the lower MMSE, the greater the degree of impairment will be. These two scores were adjusted beforehand for gender effects and for treatment doses by considering the residuals of the linear regression with gender and treatment doses as (factor and quantitative, respectively) predictors. The time scale used is patient age.

#### 6.1.2 Genetic Data

More than six million genetic markers were available after imputation for each patient. Only 2,652 of them were used, namely, those that have been associated with PD in previous studies (about 400) together with those that have an important impact on gene function (scaled CADD score ^3^ greater than 25) and an allele frequency greater than 0.01. As done classically, genetic markers with two copies of the reference allele were encoded −1, those with two copies of the alternative allele were encoded 1, and the remainder (with one copy of each) were encoded 0.

### 6.2 Results

#### 6.2.1 Model Selection Results

To ensure good interpretability of results, the number of clusters was limited to *K* = 4, and no more than two polynomial degrees were tested. The solution with the lowest BIC was obtained with four clusters and one polynomial degree.

#### 6.2.2 Clinical Results

**FIGURE 5:**
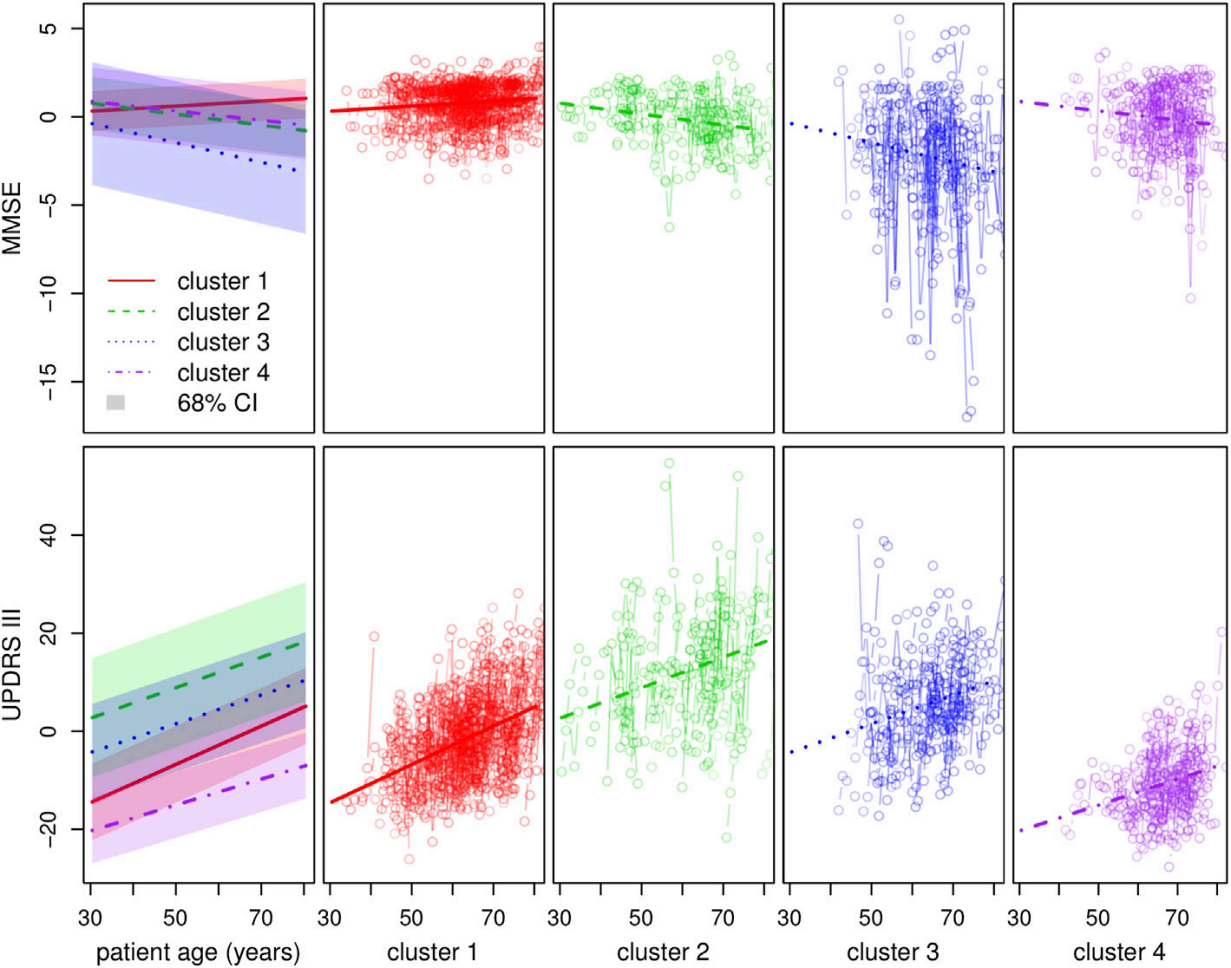
Clustering with regard to the clinical variables. The top and bottom left-hand graphs show the fitted trajectories (straight lines) for each of the four clusters and the corresponding 68% confidence intervals obtained by adding and subtracting the fitted *σ* parameters. The top *y*-axis represents the MMSE score (evaluation of cognitive impairment) and the bottom *y*-axis the UPDRS III score (motor evaluation). The other graphs show in detail, for each cluster and each score around the standard trajectory, all the trajectories of the patients assigned to the cluster.

**FIGURE 6:**
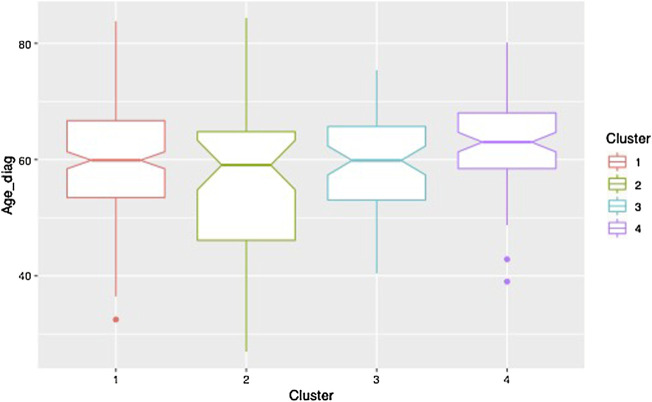
Boxplot of the age at diagnosis for each of the clusters.

Clustering results obtained from the clinical data are shown in Figure 5. Note that the variables shown here are residuals of a fitted linear model adjusted with gender and treatment doses. Patients are allocated to the cluster to which they are most likely to belong according to the model. Half of the patients are allocated to cluster 1 and the other half is allocated to three remaining clusters in approximately equal measure. The four clusters correspond to different ways in which the disease may evolve: low motor scores and no cognitive evolution (cluster 1, a mild form of PD), high motor scores and no cognitive evolution (cluster 2, a more severe motor form), high motor scores and significant cognitive evolution (clusters 3 and 4, a severe form and an intermediate form of PD). Moreover, the cluster structure is significantly related to the age of diagnosis which was not used in the clustering process. In particular, cluster 4 shows clear signs of diagnosis at a later age (Figure 6).

#### 6.2.3 Genetic Association Results

**FIGURE 7:**
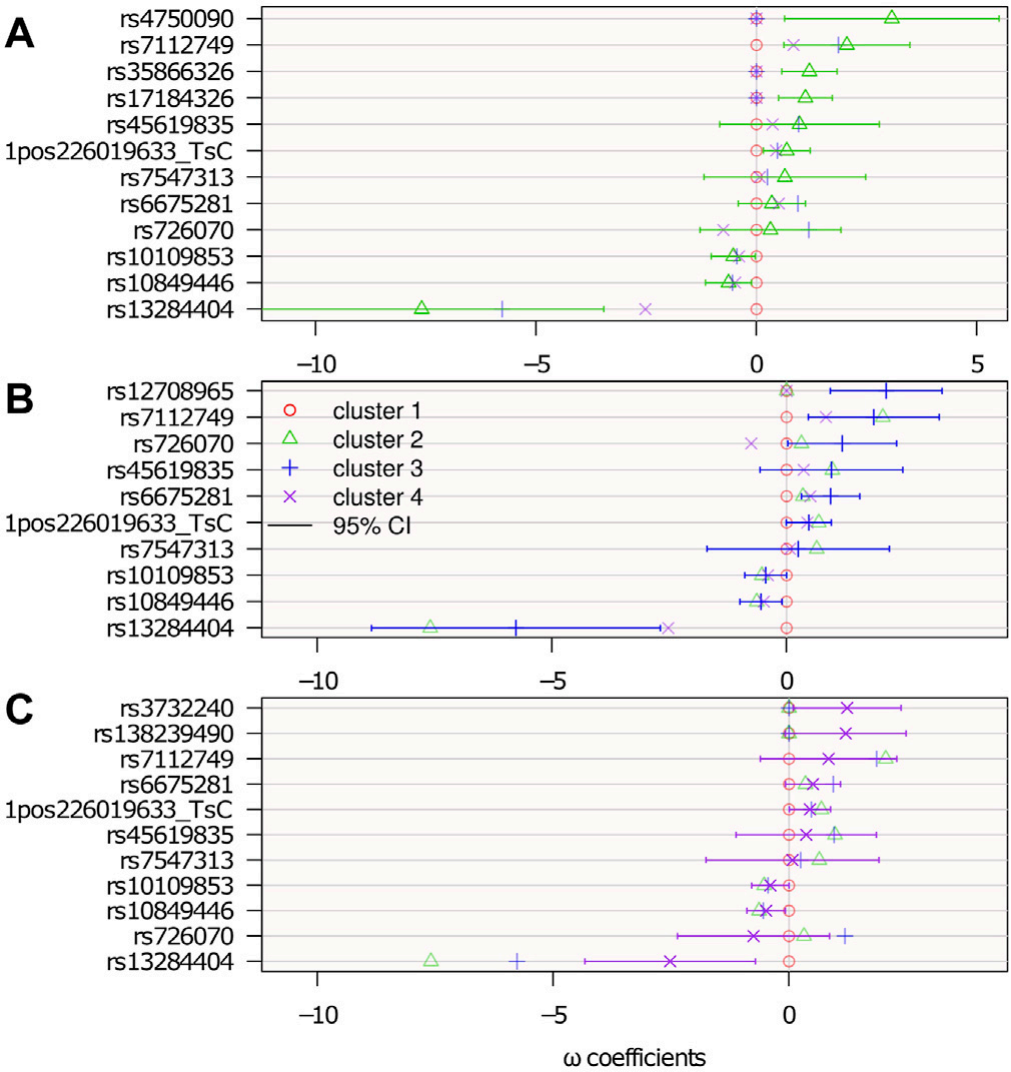
Genetic association: estimated logistic regression parameters {***ω***}. Cluster 1 is the reference: ***ω***_*ℓ*=1_ = **0**; top **(A)**: ***ω***_*ℓ*=2_; middle **(B)**: ***ω***_*ℓ*=3_; and bottom **(C)**: ***ω***_*ℓ*=4_. The confidence intervals are computed from the Hessian matrix provided by the R function nnet::nnet (Ripley et al., 2016).

Figure 7 shows the results with 95% confidence intervals linked to the parameters {***ω***} ^4^. The *p*-values below 0.05 (i.e., significant association before any multiple test correction) correspond to a 0 value outside the 95% confidence interval.

There were 15 SNPs selected, 7 of which belong to genes that potentially have a role in neurological diseases. Among the selected SNPs, rs35866326 (which appears in the ***ω***_*ℓ*=2_ panel of Figure 7) has been a focus of attention in the PD literature, having been associated with susceptibility to PD (Maraganore et al., 2005; Goris et al., 2006; Maraganore et al., 2006) although other studies (Farrer et al., 2006; Li et al., 2006) have failed to replicate this result. The lack of consensus might be because of this gene’s association only with a particular subtype of PD, as suggested in the present study, where it is associated with cluster 2 only. However, an unselected variant does not rule out any association with the disease subtype. It may be associated, but not sufficiently to contribute more information relative to the clustering.

## 7 Conclusion

### 7.1 Synthesis and Results

We proposed a model-based method for disease subtyping where the information comes from both short longitudinal data with varying observation times, as clinical follow-up data often are, and from high-dimensional quantitative data, such as genotyping data. Unlike in most multi-view clustering methods, the data are processed in a non-symmetrical way by integrating genetic data in the clustering *via* multinomial logistic weights. A Lasso penalty on the logistic regression parameters addresses the high-dimensionality of the genotyping data while exhibiting a short list of genetic factors potentially involved in the typology of the disease.

An experiment on artificial data validates our proposed inference and model selection approach and shows that it is better able to identify latent subtypes of the disease and influential genetic factors than an approach that first clusters clinical data and then performs an association study. When our method is applied on clinical and genetic data from a cohort of patients with Parkinson’s disease, we are able to characterize four distinct subtypes and 15 genetic factors with a potential impact on subtyping. Of these 15 SNPs, the most significant SNP is already associated with PD. Half of the others belong to genes suspected to be involved in neurological diseases. Being able to recover results like these shows the relevance of our approach in a real setting.

### 7.2 Perspectives

Several aspects might be revisited in future works, as outlined as follows.

#### 7.2.1 Replication

The statistical analysis presented here uses a relatively small sample size and it may thus be of interest to attempt to replicate and confirm our results using independent cohorts.

#### 7.2.2 Modeling of Data

If the objective of the subtyping is to predict the evolution of the patient’s symptoms, and if more data are available for each patient, then the temporal dynamics specific to each individual might be addressed in a more refined way, for example, using a Gaussian process as done by Schulam and Saria (2015). In addition, if the focus is on correlated clinical variables, a multivariate version of the proposed model would be interesting, but this is complicated by the functional nature of the data (*t*_*ij*_ times are different from one individual *i* to another). Regarding the genetic data, a lighter preprocessing step for the purposes of elimination may be desirable in a very high-dimensional setting (with several million SNPs), and it may consequently be useful to summarize the data, for instance, by aggregating SNPs in linkage disequilibrium blocks (Guinot et al., 2018).

#### 7.2.3 Association Study With Genetic Data

Finally, our proposed method does not dispense the need for a more traditional association study afterward, and this presents an opportunity for studying further potential associations between the genetic markers extracted in the variable selection process.

To this end, a correction for multiple testing might be done to assess the likelihood that the SNPs identified with our method actually have an impact on the disease typology. This correction should take into account the fact that the Lasso selection is performed on a large number of SNPs and that the tests are performed on a subgroup of those SNPs. Post-hoc inference tests may, therefore, be useful (Goeman and Solari, 2011).

## Data Availability

The data analyzed in this study are subject to the following licenses/restrictions: the datasets analyzed for this study belong to the APHP (Assistance Publique Hôpitaux de Paris), and can be made available upon request from J-CC. Requests to access these datasets should be directed to J-CC, jean-christophe.corvol@aphp.fr.

## Footnotes

1 For instance, clinical data may be represented by numerical scores observed on different visits (of a continuous nature with a longitudinal aspect), while genomic data may be represented by Single Nucleotide Polymorphisms (SNPs, of a categorial nature without a longitudinal aspect).

2 For instance, with the R package glmnet (Friedman et al., 2010).

3 Combined Annotation Dependent Depletion score, this score evaluates the deleteriousness of variants in the human genome (Rentzsch et al., 2018).

4 The confidence intervals are computed from the Hessian matrix provided by the R function nnet::nnet (Ripley et al., 2016).
