# Supplementary material for: A Sparse Mixture-of-Experts Model With Screening of Genetic Associations to Guide Disease Subtyping
Source: Front Genet. 2022 Jun 6;13:859462. doi: 10.3389/fgene.2022.859462 (PMC9207464; doi:10.3389/fgene.2022.859462)
Supplement: Supplementary file 1 [file Presentation1.PDF]

Supplementary Material: details of the EM Algorithm

A sparse mixture of experts models with screening of genetic associations to guide disease subtyping

1 PREAMBLE

In this section, we resume the content necessary to give the details of the calculations related to the EM algorithm of our method.

1.1 Notations of the paper

The notations and conventions are the same that those used in the paper. In particular, patients are denoted with index $i \in \{1 \cdots N\}$, variables with $v \in \{1 \cdots V\}$, visits with $j \in \{1 \cdots J\}$ and the degree of the polynomial model regarding the evolution of the symptoms with $p \in \{0 \cdots P\}$.

Remark. For the sake of clarity, we assume that all patients have the same number of visits: $\forall i, j \in \{1 \cdots J\}$. Calculations can be reproduced using the very same process given here when the number of visits per patient is different.

1.2 EM algorithm

At the $(q+1)^{\text{th}}$ iteration of the modified EM algorithm, one maximizes the expected and penalized complete-data log-likelihood $\mathcal{L}(\mathbf{Y} \mid \mathbf{G}, \mathbf{Z}; \Theta = \{\boldsymbol{\alpha}, \boldsymbol{\sigma}, \boldsymbol{\omega}\}) - \mathcal{P}(\boldsymbol{\omega})$ which reads

$$\sum_i \sum_k z_{ik} \left[\log\left[\eta_k(\mathbf{g}_i\,;\,\boldsymbol{\omega}_k)\right] + \sum_v \sum_j \log\left[f_k(y_{iv_{(j)}}\,;\,\{\boldsymbol{\alpha}_{vk}, \sigma_{vk}\}) \right] \right] - \lambda \sum_k \|\boldsymbol{\omega}_k\|_1 \,,$$

where $\lambda > 0$ controls the amount of sparsity applied on the ℓ_1 norm of $\boldsymbol{\omega}_k$ and where $\eta_k(\cdot;\cdot)$ and $f_k(\cdot;\cdot)$ are defined as follows

$$\left(y_{iv_{(j)}} \mid z_i = k\right) = \sum_p \alpha_{vkp}\, t_{ij}^p + \sigma_{vk}\, \varepsilon_{iv_{(j)}} \,, \tag{S1a}$$

$$\text{such that} \quad f_k(y_{iv_{(j)}}\,;\,\{\boldsymbol{\alpha}_{vk}, \sigma_{vk}\}) \sim \mathcal{N}\left(\sum_p \alpha_{vkp}\, t_{ij}^p,\ \sigma_{vk}^2 \right) , \tag{S1b}$$

$$\text{and} \quad \eta_k(\mathbf{g}_i\,;\,\boldsymbol{\omega}_k) = \frac{\exp\left(\omega_{k0} + \boldsymbol{\omega}_k^\mathsf{T}\mathbf{g}_i\right)}{\sum_{k'} \exp\left(\omega_{k'0} + \boldsymbol{\omega}_{k'}^\mathsf{T}\mathbf{g}_i\right)} \,, \tag{S1c}$$

To maximize the expected and penalized complete-data log-likelihood, each iteration is divided into an expectation step (E) followed by a maximization step (M).

- At step E of the $(q+1)^{\text{th}}$ iteration, posterior weights are updated as follows:

$$\tau_{ik}^{(q+1)} = \mathbb{E}\left[z_{ik}|\mathrm{Y}=\mathbf{y}_i, \mathbf{g}_i; \Theta^{(q)}\right]$$
$$= \frac{\eta_k\left(\mathbf{g}_i\,;\,\boldsymbol{\omega}_k^{(q)}\right)\prod_v\prod_j f_k\left(y_{iv_{(j)}}\,;\,\{\boldsymbol{\alpha}_{vk}^{(q)},\sigma_{vk}^{(q)}\}\right)}{\sum_{k'}\eta_{k'}\left(\mathbf{g}_i\,;\,\boldsymbol{\omega}_{k'}^{(q)}\right)\prod_v\prod_j f_{k'}\left(y_{iv_{(j)}}\,;\,\{\boldsymbol{\alpha}_{vk'}^{(q)},\sigma_{vk'}^{(q)}\}\right)}\,.$$

- At step M of the $(q+1)^{\text{th}}$ iteration, parameters are updated as follows:

$$\begin{aligned}\Theta^{(q+1)} = \underset{\Theta}{\operatorname{argmax}}\sum_i\sum_k\tau_{ik}^{(q+1)}\Bigg[&\log\left[\eta_k(\mathbf{g}_i\,;\,\boldsymbol{\omega}_k)\right]\\ &+\sum_v\sum_j\log\left[f_k(y_{iv_{(j)}}\,;\,\{\boldsymbol{\alpha}_{vk},\sigma_{vk}\})\right]\Bigg]\\ &-\lambda\sum_k\|\boldsymbol{\omega}_k\|_1\,.\end{aligned} \tag{S2}$$

2 DETAIL OF DERIVATIONS

In this section we provide the detailed expression for $\Theta^{(q+1)} = \{\boldsymbol{\alpha}_{vk}, \sigma_{vk}, \boldsymbol{\omega}_k\}$.

2.1 Additional notations

In order to reshape (S2) in a matrix form, we introduce the following notations.

- Let $\mathbf{y}_v = \left(y_{1v_{(1)}}, \cdots, y_{iv_{(1)}}, \cdots, y_{iv_{(J)}}, \cdots, y_{Nv_{(J)}}\right)^{\mathsf{T}}$ be the vector of size NJ which gathers the measurements of the variable v for all patients $i \in \{1\cdots N\}$ and all visits $j \in \{1\cdots J\}$,
- Let $\mathbf{T}$ be the matrix of size $(NJ)\times P+1$ which gathers all vectors $\mathbf{t}_{ij} = \left(1, t_{ij}, \cdots, t_{ij}^{P-1}, t_{ij}^{P}\right)$ for the patient i at visit j, regarding all degrees $p \in \{0\cdots P\}$,
- Let $\mathbf{W}_k = \operatorname{diag}(\underbrace{\tau_{1k}\cdots\tau_{1k}}_{J\text{ times}}, \cdots, \underbrace{\tau_{Nk}\cdots\tau_{Nk}}_{J\text{ times}})$ be the diagonal matrix of size $(NJ)\times(NJ)$ which gathers the probabilities τ_{ik} of all patients to belong to class k.

2.2 Optimization of clinical parameters $\{\boldsymbol{\alpha}_{vk}, \boldsymbol{\sigma}_{vk}\}$

Equation (S2) involves $f_k(y_{iv_{(j)}}\,;\, \{\boldsymbol{\alpha}_{vk}, \sigma_{vk}\})$ which is a Gaussian distribution defined in (S1b). Regarding the parameters $\{\boldsymbol{\alpha}_{vk},\, \sigma_{vk}\}$, Equation (S2) reads

$$
\begin{aligned}
\{\boldsymbol{\alpha}_{vk}^{(q+1)},\, \sigma_{vk}^{(q+1)}\} = \underset{\{\boldsymbol{\alpha}_{vk},\, \sigma_{vk}\}}{\operatorname{argmax}} & \sum_i \tau_{ik}^{(q+1)} \sum_j \log\left[f_k(y_{iv_{(j)}}\,;\, \{\boldsymbol{\alpha}_{vk}, \sigma_{vk}\})\right] \\
& \sum_i \sum_j \tau_{ik}^{(q+1)} \log\left[\frac{1}{\sqrt{2\pi}\sigma_{vk}} \exp\left(-\frac{1}{2\,\sigma_{vk}^2}\left(y_{iv_{(j)}} - \sum_p \alpha_{vkp}\, t_{ij}^p\right)^2\right)\right] \\
& \sum_i \sum_j \tau_{ik}^{(q+1)} \left[-\log(\sigma_{vk}) - \frac{1}{2\,\sigma_{vk}^2}\left(y_{iv_{(j)}} - \sum_p \alpha_{vkp}\, t_{ij}^p\right)^2\right] . \qquad \text{(S3)}
\end{aligned}
$$

2.2.1 Optimization of $\{\boldsymbol{\alpha}_{vk}\}$

Regarding the parameters $\{\boldsymbol{\alpha}_{vk}\}$, Equation (S3) reads

$$
\begin{aligned}
\boldsymbol{\alpha}_{vk}^{(q+1)} = \underset{\boldsymbol{\alpha}_{vk}}{\operatorname{argmax}} & \sum_i \sum_j \tau_{ik}^{(q+1)} \left(y_{iv_{(j)}} - \sum_p \alpha_{vkp}\, t_{ij}^p\right)^2 \\
& \sum_i \sum_j \tau_{ik}^{(q+1)} \left\| y_{iv_{(j)}} - \mathbf{t}_{ij}\, \boldsymbol{\alpha}_{vk} \right\|^2 \\
& (\mathbf{y}_v - \mathbf{T}\,\boldsymbol{\alpha}_{vk})^\mathsf{T}\, \mathbf{W}\, (\mathbf{y}_v - \mathbf{T}\,\boldsymbol{\alpha}_{vk}) \ .
\end{aligned}
$$

Let $g(\boldsymbol{\alpha}_{vk}) = (\mathbf{y}_v - \mathbf{T}\,\boldsymbol{\alpha}_{vk})^\mathsf{T}\, \mathbf{W}\, (\mathbf{y}_v - \mathbf{T}\,\boldsymbol{\alpha}_{vk})$. The optimality condition for $\boldsymbol{\alpha}_{vk}^{(q+1)}$ is reached when

$$
\begin{aligned}
\frac{\partial g(\boldsymbol{\alpha}_{vk})}{\partial \boldsymbol{\alpha}_{vk}} = 0 \quad & \Longleftrightarrow \quad 2\,\mathbf{T}^\mathsf{T}\mathbf{W}\mathbf{T}\boldsymbol{\alpha}_{vk} - 2\,\mathbf{T}^\mathsf{T}\mathbf{W}\mathbf{y}_v = 0 \\
& \Longleftrightarrow \quad (\mathbf{T}^\mathsf{T}\mathbf{W}\mathbf{T})^{-1}\,\mathbf{T}^\mathsf{T}\mathbf{W}\mathbf{y}_v = \boldsymbol{\alpha}_{vk}^{(q+1)} \ .
\end{aligned}
$$

2.2.2 Optimization of $\{\boldsymbol{\sigma}_{vk}\}$

Regarding the parameters $\{\sigma_{vk}\}$, let $h(\sigma_{vk})$ be Equation (S3), that is

$$
h(\sigma_{vk}) = \sum_i \sum_j \tau_{ik}^{(q+1)} \left[-\log(\sigma_{vk}) - \frac{1}{2\,\sigma_{vk}^2}\left(y_{iv_{(j)}} - \sum_p \alpha_{vkp}\, t_{ij}^p\right)^2\right] .
$$

The optimality condition for $\sigma_{vk}^{(q+1)}$ is reached when

$$
\begin{aligned}
\frac{\partial h(\sigma_{vk})}{\partial \sigma_{vk}^2} = 0 \quad &\Longleftrightarrow \quad \sum_i \sum_j \tau_{ik}^{(q+1)} \left(-\frac{1}{2\sigma_{vk}^2} + \frac{1}{2\sigma_{vk}^4} \left(y_{iv_{(j)}} - \sum_p \alpha_{vkp}\, t_{ij}^p \right)^2 \right) &&= 0 \\
&\Longleftrightarrow \quad \frac{\sum_i \sum_j \tau_{ik}^{(q+1)} \left(y_{iv_{(j)}} - \sum_p \alpha_{vkp}\, t_{ij}^p \right)^2}{\sigma_{vk}^2} &&= \sum_i \sum_j \tau_{ik}^{(q+1)} \\
&\Longleftrightarrow \quad \frac{\sum_i \sum_j \tau_{ik}^{(q+1)} \left(y_{iv_{(j)}} - \sum_p \alpha_{vkp}\, t_{ij}^p \right)^2}{\sum_i \sum_j \tau_{ik}^{(q+1)}} &&= \sigma_{vk}^{2\ (q+1)} .
\end{aligned}
$$

Finally,

$$
\sigma_{vk}{}^{(q+1)} = \sqrt{\frac{\sum_i \sum_j \tau_{ik}^{(q+1)} \left(y_{iv_{(j)}} - \sum_p \alpha_{vkp}\, t_{ij}^p \right)^2}{\sum_i \sum_j \tau_{ik}^{(q+1)}}} .
$$

2.3 Optimization of genetic parameters $\{\boldsymbol{\omega}_k\}$

Equation (S2) involves $\eta_k(\mathbf{g}_i\,;\,\boldsymbol{\omega}_k)$ which comes from the multinomial logistic model defined in (S1c). Regarding the parameters $\{\boldsymbol{\omega}_k\}$, Equation (S2) reads

$$
\begin{aligned}
\boldsymbol{\omega}_k^{(q+1)} = \underset{\boldsymbol{\omega}_k}{\operatorname{argmax}} & \sum_i \sum_k \tau_{ik}^{(q+1)} \Big[\log \left[\eta_k(\mathbf{g}_i\,;\,\boldsymbol{\omega}_k) \right] \Big] && - \lambda \sum_k \|\boldsymbol{\omega}_k\|_1 \\
& \sum_i \sum_k \tau_{ik}^{(q+1)} \left[\log \left[\frac{\exp\left(\omega_{k0} + \boldsymbol{\omega}_k^\intercal \mathbf{g}_i\right)}{\sum_{k'} \exp\left(\omega_{k'0} + \boldsymbol{\omega}_{k'}^\intercal \mathbf{g}_i\right)} \right] \right] && - \lambda \sum_k \|\boldsymbol{\omega}_k\|_1 \\
& \sum_i \sum_k \tau_{ik}^{(q+1)} \left[(\omega_{k0} + +\boldsymbol{\omega}_k^\intercal) - \log \left[\sum_{k'} \exp\left(\omega_{k'0} + \boldsymbol{\omega}_{k'}^\intercal \mathbf{g}_i\right) \right] \right] && - \lambda \sum_k \|\boldsymbol{\omega}_k\|_1 .
\end{aligned}
$$

The above expression corresponds to the maximisation problem related to Equation (4) in the paper. The monograph of Hastie et al. (2015) specifically address the resolution of the ℓ_1-penalized multinomial logistic regression problem in Section 3.3 with the corresponding algorithm based on a proximal-Newton approach in Section 3.3.2, Section 3.7 and Section 5.3.3. The papers of Polson et al. (2015) and Parikh and Boyd (2014) are also useful resources for understanding proximal algorithms.

REFERENCES

Hastie, T., Tibshirani, R., and Wainwright, M. (2015). Statistical learning with sparsity. *Monographs on statistics and applied probability* 143, 143

Parikh, N. and Boyd, S. (2014). Proximal algorithms. *Foundations and Trends in optimization* 1, 127–239

Polson, N. G., Scott, J. G., and Willard, B. T. (2015). Proximal algorithms in statistics and machine learning. *Statistical Science* 30, 559–581
